# A putative exosporium lipoprotein GBAA0190 of *Bacillus anthracis* as a potential anthrax vaccine candidate

**DOI:** 10.1186/s12865-021-00414-y

**Published:** 2021-03-21

**Authors:** Jun Ho Jeon, Yeon Hee Kim, Kyung Ae Kim, Yu-Ri Kim, Sun-Je Woo, Ye Jin Choi, Gi-eun Rhie

**Affiliations:** Division of High-risk Pathogens, Bureau of Infectious Disease Diagnosis Control, Korea Disease Control and Prevention Agency, Cheongju, 28159 Republic of Korea

**Keywords:** *Bacillus anthracis*, Anthrax, Vaccine, Exosporium lipoprotein

## Abstract

**Background:**

*Bacillus ancthracis* causes cutaneous, pulmonary, or gastrointestinal forms of anthrax*. B. anthracis* is a pathogenic bacterium that is potentially to be used in bioterrorism because it can be produced in the form of spores. Currently, protective antigen (PA)-based vaccines are being used for the prevention of anthrax, but it is necessary to develop more safe and effective vaccines due to their prolonged immunization schedules and adverse reactions.

**Methods:**

We selected the lipoprotein GBAA0190, a potent inducer of host immune response, present in anthrax spores as a novel potential vaccine candidate. Then, we evaluated its immune-stimulating activity in the bone marrow-derived macrophages (BMDMs) using enzyme-linked immunosorbent assay (ELISA) and Western blot analysis. Protective efficacy of GBAA0190 was evaluated in the guinea pig (GP) model.

**Results:**

The recombinant GBAA0190 (r0190) protein induced the expression of various inflammatory cytokines including tumor necrosis factor-α (TNF-α), interleukin-6 (IL-6), monocyte chemoattractant protein-1 (MCP-1), and macrophage inflammatory protein-1α (MIP-1α) in the BMDMs. These immune responses were mediated through toll-like receptor 1/2 via activation of mitogen-activated protein (MAP) kinase and Nuclear factor-κB (NF-κB) pathways. We demonstrated that not only immunization of r0190 alone, but also combined immunization with r0190 and recombinant PA showed significant protective efficacy against *B. anthracis* spore challenges in the GP model.

**Conclusions:**

Our results suggest that r0190 may be a potential target for anthrax vaccine.

**Supplementary Information:**

The online version contains supplementary material available at 10.1186/s12865-021-00414-y.

## Background

*Bacillus anthracis* is a Gram-positive, spore-forming, rod-shaped bacterium which causes acute infectious disease anthrax. In nature, vegetative bacilli can transform to dormant spores which survive harsh environment for long periods [1]. Because *B. anthracis* can be produced in the form of spores, it is highly likely to be used as a bioterrorism agent, so it is designated as a tier 1 biological select agent by the US Centers for Disease Control and Prevention [2]. After entering the host through the routes of lung, skin, or gastrointestinal tract, *B. anthracis* spores germinate and multiply without being completely scavenged by host immune cells [1].

Antigen-presenting cells (APC) including macrophages and dendritic cells are known to be reservoirs for spores to germinate within the cells [3]. Once escaped from the APC, anthrax bacilli multiply rapidly and spread systemically via the bloodstream, secreting high levels of exotoxins [4, 5]. Toxins are composed of three distinct proteins: protective antigen (PA), edema factor (EF), and lethal factor (LF) [6]. The binding of PA to LF or EF forms lethal toxin (LT) and edema toxin (ET), respectively [7]. LT is a zinc-dependent metalloprotease leading to the inactivation of mitogen-activated protein (MAP) kinase kinases, which play an important role in cellular responses to a variety of stimuli such as proinflammatory cytokines and cellular stresses [4]. ET works as a calmodulin-dependent adenylate cyclase that elevates intracellular level of cyclic adenosine monophosphate (cAMP) resulting in a severe change of cellular water balance and producing massive edema [4, 5]. Among these three proteins, PA has been the primary target for developing or licensed anthrax vaccines such as anthrax vaccine adsorbed (AVA) and anthrax vaccine precipitated (AVP) [8].

Bacterial lipoproteins possess an N-terminal signal sequence in which a positively charged region and a conserved lipobox motif exist [9]. The consensus sequence of lipobox is [LVI] [ASTVI][GAS][C] [9]. Diacylglycerol moiety is covalently attached to the thiol group of the conserved cysteine residue by the prolipoprotein diacylglyceryl transferase (Lgt), leading to the formation a prolipoprotein [10]. Then, the third acyl chain is attached to the cysteine residue of the signal peptide-cleaved lipoprotein by amide linkage [10]. Mature lipoprotein interacts with toll-like receptor (TLR) 2 forming a heterodimeric complex with either TLR1 or TLR6, resulting in stimulating host innate immune responses [11]. Bacterial lipoproteins play a crucial role in growth, virulence, and survival of bacteria [10, 12]. Indeed, the *B. anthracis lgt* gene mutant strain showed impaired germination ability and reduced virulence in the mouse model of spore infection [12]. Furthermore, the *lgt* mutant induced lower inflammatory responses in the mouse macrophages, and these responses were not TLR2-dependent [12]. Currently, bacterial lipoproteins are being developed as vaccine candidates or adjuvants for the prevention of various infectious diseases [10, 11, 13].

Although the PA-based vaccines for anthrax prophylaxis are currently available, various efforts have been made to improve vaccine efficacy or reduce immunization schedule. The PA plus formaldehyde-inactivated spores of *B. anthracis* (PA-FIS) showed a better protection in the inhalation model of anthrax than the PA alone [14]. Additionally, immunization of PA-LF chimera antigen provided more effective protection against *B. anthracis* spore challenge than that with PA83 alone [15]. Single-dose adenovirus type 5-vector and AV7909 anthrax vaccines were being developed to reduce the immunization schedule of the AVA vaccine [16–18].

In this study, we selected the GBAA0190 protein, which is a putative lipoprotein located on the spores of *B. anthracis* and its closely related species such as *Bacillus cereus* and *Bacillus thuringiensis* [19–21]. Then we examined immunostimulating activity of the GBAA0190 (r0190) in the bone marrow-derived macrophages (BMDMs) and evaluated the protective efficacy of the r0190 against *B. anthracis* spore challenge in the guinea pig (GP) model.

## Results

### GBAA0190 expression in the exosporium of *B. anthracis* H9401

**Fig. 1:**
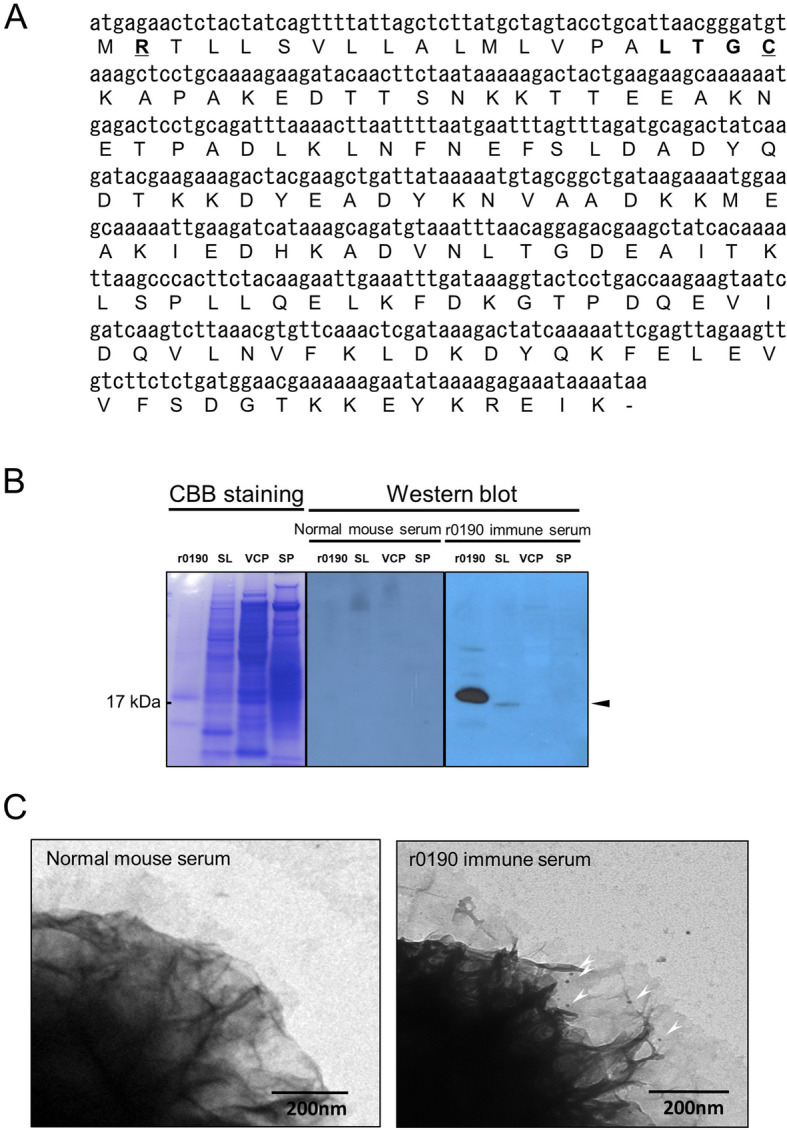
GBAA0190 protein expression in the surface of *B. anthracis* spore. **a** The Nucleotide and amino acid sequences of GBAA0190. The positively charged region and lipobox were shown in bold, whereas the positively charged region and cysteine residue were underlined. **b** GBAA0190 protein was specifically expressed in *B. anthracis* spores. Protein fractions such as spore lysates (SL, 30 μg), vegetative cell proteins (VCP, 30 μg), and secreted proteins (SP, 30 μg) of *B. anthracis* H9401 and r0190 (1 μg) were analyzed by SDS-PAGE, followed by staining with coomassie brilliant blue (CBB) and Western blotting with normal mouse serum or r0190 immune serum (1: 1000 dilution). **c** Detection of GBAA0190 protein on *B. anthracis* H9401 spore exosporium. Spores were treated with fixation solution and incubated with r0190 immune serum (1:1000 dilution), followed by incubation with 10 nm gold-conjugated secondary antibodies (black dots)

Bacterial lipoprotein is a potent inducer of immune responses and has attracted attention as a vaccine candidate. It has been reported that 138 lipoprotein candidates are present in *B. anthracis* genome [12]. Of the various lipoproteins, the GBAA0190 protein was selected as a vaccine target because it is located on the *B. anthracis* spores [22] that make initial contacts with the host immune system. GBAA0190 is a 156 amino acid protein and the gene is located in the chromosome of *B. anthracis* Ames ancestor from 191,142 to 191,609 [23], whose function is not characterized. Like other bacterial lipoproteins, GBAA0190 coding sequences contain signal sequence, lipobox, and a crucial cysteine residue that is modified by covalent attachment of diacylglycerol moiety, based on the DOLOP database (http://www.mrc-lmb.cam.ac.uk) (Fig. 1a) [9]. The protein coding region of GBAA0190 was cloned into the pET19b expression plasmid. Then the protein expression was analyzed by sodium dodecyl sulfate-polyacrylamide gel electrophoresis (SDS-PAGE) and following Western blot analysis using antiserum against GBAA0190. To examine whether the GBAA0190 protein is actually expressed in the *B. anthracis* spores, proteins were prepared from spores, vegetative cells, and culture supernatants, respectively. Western blot analysis showed that the GBAA0190 protein was expressed only in anthrax spores at approximately 17 kDa (Fig. 1b). In addition, immunoreactive band was also detected for the r0190 (Fig. 1b). To further demonstrate whether the GBAA0190 protein is localized in anthrax spores, we conducted immunoelectron microscopy using antiserum against r0190. As shown in Fig. 1c, r0190 antiserum specifically bound to exosporium of *B. anthracis*, whereas control serum did not. These results indicate that GBAA0190 is a spore protein of *B. anthracis*.

### Cytokine production by r0190 in BMDMs

**Fig. 2:**
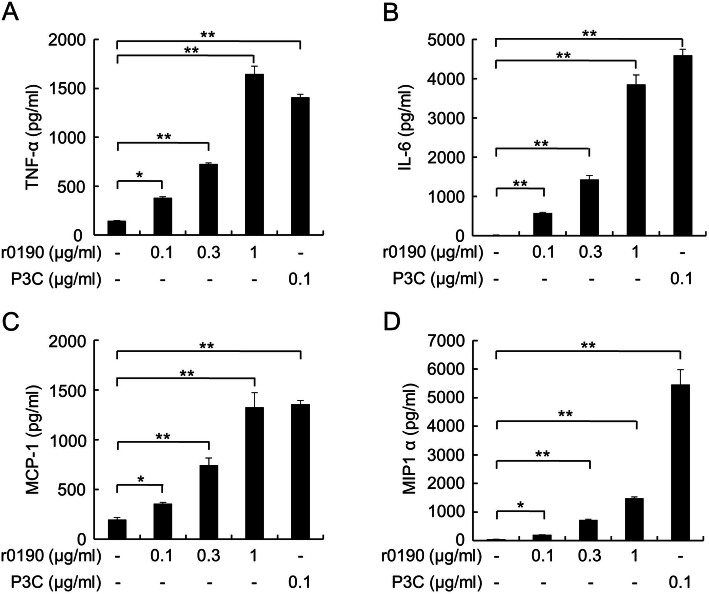
r0190-induced cytokine production. BMDMs from C57BL/6 mice were stimulated with r0190 (0.1, 0.3, or 1 μg/ml) or P3C (0.1 μg/ml) for 24 h. At the end of the incubation period, the level of TNF-α, IL-6, MCP-1, and MIP-1α was measured by ELISA.* *P* < 0.05, ** *P* < 0.01 as compared with the non-treatment control group

Because bacterial lipoprotein is known to be an effective inducer of immune response, we next investigated whether r0190 stimulates expression of proinflammatory cytokines in mouse BMDMs. Treatment of the cells with r0190 significantly enhanced expression of tumor necrosis factor-α (TNF-α), interleukin (IL)-6, monocyte chemoattractant protein-1 (MCP-1), and macrophage inflammatory protein-1α (MIP1α) in a dose-dependent manner (Fig. 2a-d).

### Involvement of TLR2 and TLR1 in r0190-induced inflammatory response

**Fig. 3:**
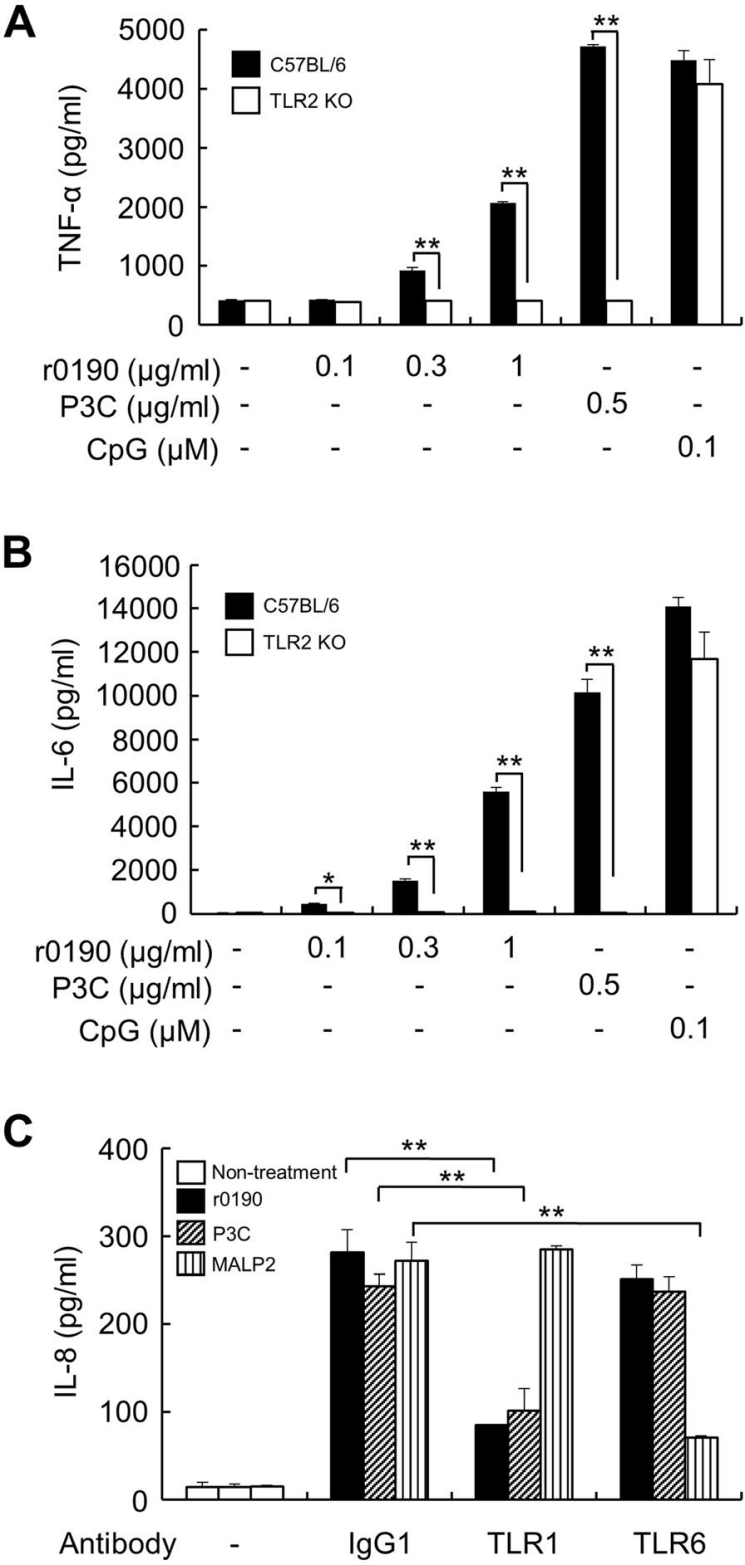
Involvement of TLR2 and TLR1 in r0190-induced inflammatory response. BMDMs from C57BL/6 and TLR2-KO C57BL/6 mice were stimulated with r0190 (0.1, 0.3, or 1 μg/ml), P3C (0.5 μg/ml), or CpG 2395 (0.1 μΜ) for 24 h. The level of TNF-α (**a**) and IL-6 (**b**) were measured by ELISA. **c** HEK293-TLR2 cells were pretreated with control IgG1 (5 μg/ml), α-TLR1 (5 μg/ml), or α-TLR6 (5 μg/ml) for 1 h, followed by stimulation with r0190 (1 μg/ml), P3C (0.1 μg/ml), or MALP2 (0.01 μg/ml) for an additional 24 h. Cell culture media were then collected and IL-8 concentration was measured by ELISA. .* *P* < 0.05, ** *P* < 0.01 as compared with the appropriate control group

Because bacterial lipoprotein induces an immune response through TLR2, we investigated whether r0190 induces TLR2-dependent cytokine expression. BMDMs from C57BL/6 wild-type (WT) and TLR2-knockout (KO) were treated with r0190, PAM3CSK4 (P3C), or CpG2395 (CpG) DNA for 24 h. As shown in Fig. 3a and b, r0190-induced expression of TNF-α and IL-6 was completely abolished in TLR2-deficient BMDMs. Similar observation was found in P3C-treated cells, whereas such effect was not observed in CpG DNA-treated cells. TLR2 forms a heterodimer with either TLR1 or TLR6 to induce an immune response upon ligand binding. Thus, we examined whether r0190 can elicit an inflammatory response through TLR1/TLR2 or TLR2/TLR6. HEK293-TLR2 cells were pretreated with neutralizing antibodies against TLR1 or TLR6 or isotype control antibody for 1 h, and then treated with r0190, P3C, or macrophage-activating lipopeptide2 (MALP2) for 24 h. TLR1 neutralizing antibody significantly attenuated IL-8 production by r0190 and P3C but did not inhibit MALP2-induced IL-8 production (Fig. 3c). On the other hand, TLR6 neutralizing antibody reduced IL-8 production only in MALP2-treated cells but not in r0190- and P3C-stimulated cells (Fig. 3c). These data imply that r0190 is a ligand for TLR1/TLR2.

### Cytokine production by r0190 via MAP kinase and NF-κB pathways

**Fig. 4:**
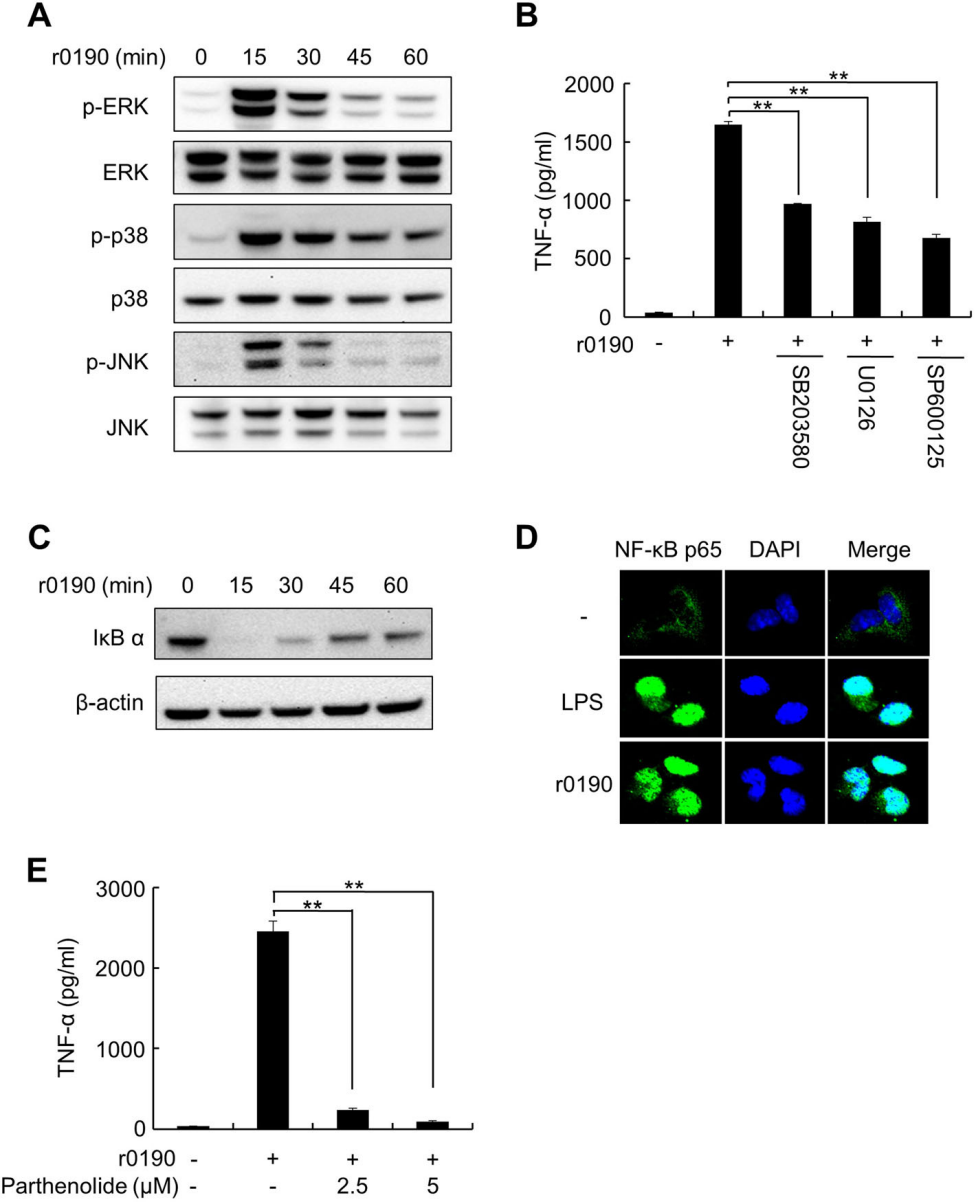
Cytokine production by r0190 via MAP kinase and NF-κB pathways. **a** BMDMs were treated with r0190 (1 μg/ml) for the indicated time periods. At the end of the stimulation periods, the cells were lysed, and cell lysates were subjected to Western blot analysis to determine the intracellular levels of phosphorylated or unphosphorylated forms of ERK, p38, and JNK. **b** BMDMs were pretreated with the indicated concentrations of MAP kinase inhibitors including ERK (U0126), p38 (SB203580), or JNK (SP600125) for 1 h, followed by stimulation with r0190 (1 μg/ml) for an additional 24 h. Secreted TNF-α in culture supernatant was quantified by ELISA. Values are the mean ± SD of triplicate samples. ** *P* < 0.01 as compared with the r0190-treated group. **c** BMDMs were treated with r0190 (1 μg/ml) for the indicated time periods. At the end of the stimulation periods, the cells were lysed, and cell lysates were subjected to Western blot analysis to determine the intracellular levels of IκB α. **d** BMDMs were stimulated with r0190 (1 μg/ml) for 1 h and translocation of p65 was determined by immunofluorescence staining with anti-p65 antibody, followed by Alexa-488–conjugated secondary antibody and DAPI. Images were obtained by Olympus FV1000 confocal microscope. **e** BMDMs were pretreated with the indicated concentrations of parthenolide for 1 h and then stimulated with r0190 (1 μg/ml) for an additional 24 h. TNF-α expression was measured by ELISA. Values are the mean ± SD of triplicate samples. ** *P* < 0.01 as compared with the r0190-treated group

Because MAP kinase and Nuclear factor-κB (NF-κB) signaling pathways play an important role in the TLR2-mediated cytokine expression [24], we examined whether r0190 can induce the activation of these signaling molecules in BMDMs. Stimulation of the cells with r0190 robustly increased phosphorylation of the MAP kinases ERK, p38, and JNK at 15 min and this activation declined thereafter (Fig. 4a). To determine whether the enhancement of the MAP kinases activation is involved in r0190-mediated cytokine expression in BMDMs, the cells were pretreated with inhibitors against MAP kinases followed by stimulation with r0190. As shown in Fig. 4b, all three inhibitors for MAP kinases attenuated the r0190-induced TNF-α expression. Next, we examined whether NF-κB signaling pathways are involved in r0190-mediated cytokine expression in BMDMs using Western blotting and confocal microscopy. As shown in Fig. 4c and d, stimulation of the cells with r0190 maximally induced not only degradation of I-κB-α, an inhibitor of NF-κB transcription factor after stimulation for 15 min, but also nuclear translocation of NF-κB p65 protein after stimulation for 1 h. Additionally, NF-κB inhibitor, parthenolide, significantly attenuated r0190-mediaed TNF-α expression in BMDMs (Fig. 4e). These results indicate that MAP kinase and NF-κB signaling pathways play a pivotal role in r0190-mediated cytokine expression.

### Protective efficacy of r0190 in the GPs

**Fig. 5:**
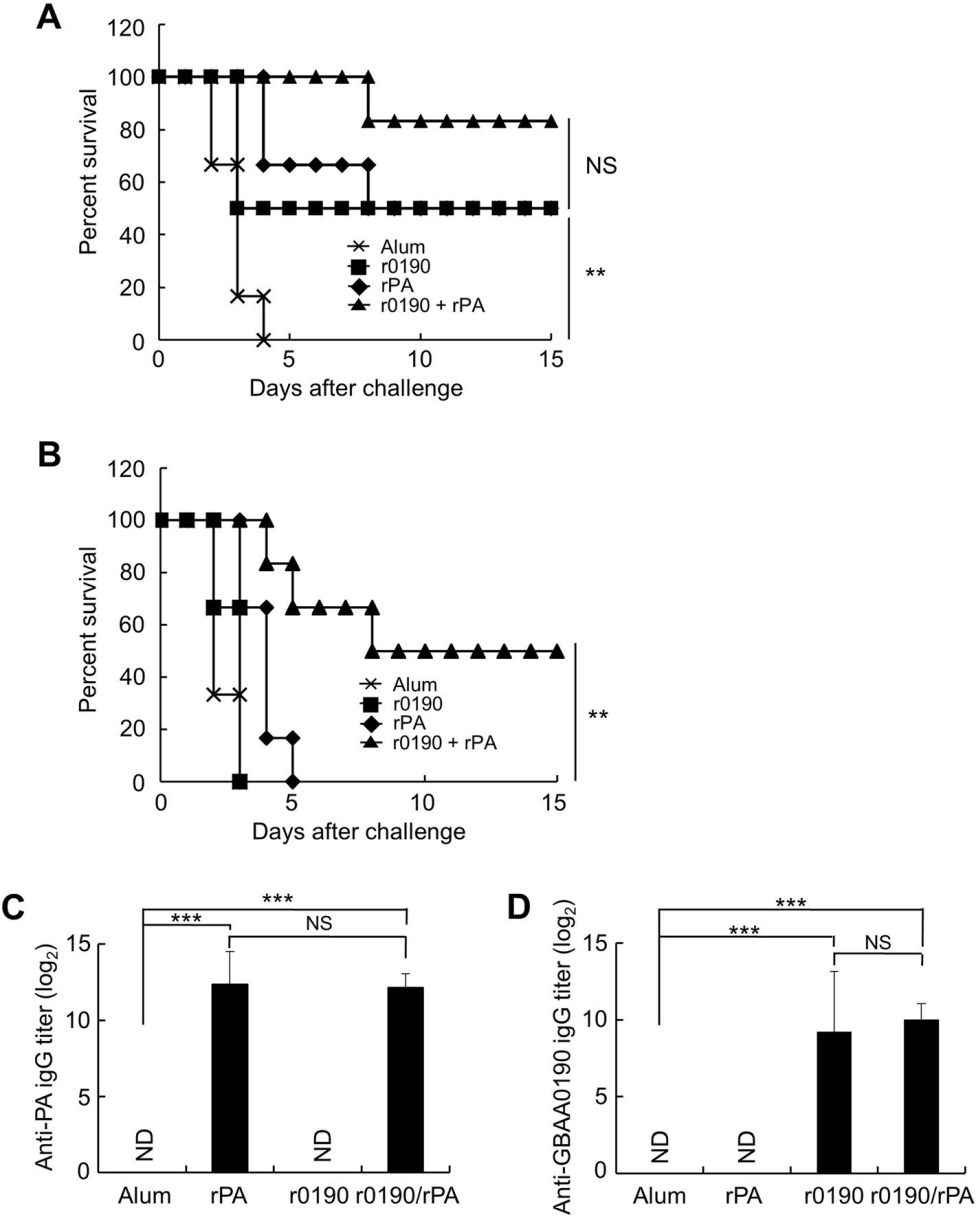
Protective efficacy of r0190 against *B. anthracis* H9401 spore challenge. Survival of GPs against 15 (**a**) or 30 (**b**) LD_50_ of *B*. *anthracis* H9401 challenge. GPs were immunized with r0190 (5 μg), rPA (5 μg) or a mixture of r0190 and rPA (5 μg each) by i.m. injection on days 0 and 28. Alum was used as a control. The protective efficacy was evaluated by challenging with 15 or 30 LD_50_ of *B*. *anthracis* H9401 by i.m. injection on day 28 after the final vaccination. Animals that survived for 14 days were considered survivors. ** *P* < 0.01 as compared with the control group. **c**, **d** Antigen-specific IgG antibody titers against PA or GBAA0190 were determined by ELISA. Groups were compared by multiple-comparison test after one-way ANOVA. *** *P* < 0.001 as compared with the alum-immunized group. ND, not detected. NS, not significant

Since we confirmed that the r0190 can induce activation of immune cells, we next evaluated its protective efficacy as a potential vaccine target in the GP model. Groups of GPs (*n* = 6) were immunized by i.m. route on days 0 and 28 with r0190 alone (5 μg), rPA alone (5 μg), or a mixture of r0190 and rPA (r0190 + rPA, 5 μg of each) in alum, followed by i.m. challenge with 15 LD_50_ of *B. anthracis* spores (751 spores) on day 56. All the aluminum hydroxide (alum)-immunized GPs succumbed to anthrax 3–4 days postinfection, while r0190 or rPA protected 50% of the challenged GPs and 83% of those immunized with r0190 plus rPA were protected against *B. anthracis* spore challenge (Fig. 5a). Next, we evaluated the protective efficacy of the same antigens as above when GPs were challenged with 30 LD_50_ of *B. anthracis* spores (1503 spores). As shown in Fig. 5b, immunization of r0190, rPA, or alum alone failed to protect *B. anthracis* spore-challenged GPs. Nevertheless, 50% of the challenged GPs were protected by the combination of r0190 and rPA (*P* < 0.01). Additionally, we assessed immunogenicity of immunized proteins by ELISA using sera collected from mice 28 days after the second immunization. Mouse groups immunized with rPA alone or a mixture of rPA and r0190 exhibited significantly higher anti-PA antibody titers compared with the group immunized with alum only (*P* < 0.001) (Fig. 5c). Similar to the result of the PA ELISA, anti-GBAA0190 antibody titers were significantly increased in r0190 or r0190 plus rPA-immunized groups compared with the alum-immunized group (P < 0.001) (Fig. 5d). These results imply that r0190 may be a potential component of multivalent anthrax vaccine.

## Discussion

In the present study, we reported the recombinant GBAA0190 protein is a vaccine candidate that can activate protective immune responses against anthrax spores. We found that the GBAA0190 protein was located in the exosporium of *B. anthracis* and the r0190 protein was able to induce protection against anthrax spores when immunized alone or with PA in the GP model. Furthermore, we demonstrated that r0190 protein is a TLR1/TLR2 ligand that activates intracellular signaling molecules including MAP kinases and NF-κB, thereby inducing inflammatory responses.

Various bacterial lipoproteins have been developed as vaccine candidates for the prevention of infectious diseases. In fact, Immunization of the lipoprotein CD0873 protected from gut colonization by *Clostridium difficile* in the mouse model [25]. The lipoprotein NMB0928 showed protective immunity against group B *Neisseria meningitidis* infection in the animal model [26]. The group B meningococcal Trumenba lipoprotein vaccine has been approved for protection against group B meningococcal disease in the various countries [27]. Intranasal immunization with lipoproteins and cholera toxin subunit B provided protection from *Streptococcus pneumoniae* colonization [28].

Although the function of GBAA0190 is not known, protective capacity of the r0190 protein against *B. anthracis* spore challenge may be due to enhanced immune responses through TLR1 and TLR2. In fact, licensed meningococcal meningitis B vaccine rLP2086s augmented secreted alkaline phosphatase activity regulated by NF-κB in the HEK293 TLR2 cells, which expresses TLR1, and these enhancements were reduced by anti-TLR2 monoclonal antibody [29]. In addition, immunization of *Borrelia burgdorferi* OspA induced low titers of OspA antibodies in the TLR2 or TLR1-deficient mice compared with those in the wild-type mice [30]. *Bordetella pertussis* lipoprotein BP1569 enhanced TNF-α production in the TLR4-deficient dendritic cells and this effect was abrogated by anti-TLR2 antibody [31]. In this study, r0190-mediated cytokine secretion was not found in TLR2-deficient BMDMs. In addition, TLR1 blocking antibody attenuated r0190-mediated IL-8 expression in HEK293 TLR2 cells.

The exosporium is the outermost layer of *B. anthracis* and contains various proteins including BclA, CotY, BxpB, and GroEL [32, 33]. These proteins have attracted much attention as anthrax vaccine candidates since they firstly contact with host innate immune system during the early phases of infection. Indeed, the recombinant BclA enhanced rPA-induced protection against *B. anthracis* spore challenge in the A/J mouse model [34]. Additionally, immunization of the recombinant GroEL augmented the rPA-mediated protective immune response against *B. anthracis* spore challenge in the BALB/c mouse model [35].

## Conclusion

In this study, we demonstrated that combined immunization of r0190 and rPA induced significant protection against anthrax spore challenge in the GP model. Our current study is the first to demonstrate that the putative lipoprotein r0190 may be a potential target for multivalent anthrax vaccines.

## Methods

### Reagents and chemicals

All MAP kinase inhibitors, including SB203580, U0126, and SP600125, were obtained from Calbiochem (Darmstadt, Germany). Parthenolide, an NF-κB inhibitor, was purchased from Sigma-Aldrich Chemical Inc. (St. Louis, MO. USA). Antibodies specific for IκB-α, β-actin, and MAP kinases were from Cell Signaling Technology (Beverly, MA, USA). TLR1 and TLR6 neutralizing antibodies, P3C and CpG were purchased from InvivoGen (Cayla SAS, Toulouse, France). MALP2 was obtained from Alexis Biochemicals (San Diego, CA, USA). Enzyme-linked immunosorbent assay (ELISA) kits for TNF-α, IL-6, MCP-1, and IL-8 were purchased from Biolegend (San Diego, CA, USA) and MIP-1α ELISA kit was obtained from R&D systems (Minneapolis, MN, USA). Aluminum.

### Animals

Six-week-old female C57BL/6 WT mice were purchased from Samtako (Osan, Korea). TLR2-KO female C57BL/6 mice were obtained from Jackson laboratory (Bar Harbor, ME, USA). Five-week-old female GPs were purchased from Samtaco (Osan, Korea). Mice and GPs were housed in the specific-pathogen-free animal facility. All experiments were conducted in compliance with the guidelines of the Korea Disease Control and Prevention Agency Institutional Animal Care and Use Committee (Approval number: KCDC-129-18-2A).

### Recombinant protein expression of GBAA0190

The GBAA0190 gene was PCR-amplified from *B. anthracis* ATCC 14578 genomic DNA using specific primers with *Nde* I and *Xho* I restriction sites. The primers were as follows: forward primer 5′-GGCATATGAGAACTCTACTATCA-3′ and reverse primer 5′-GGCTCGAGTTATTTTATTTCTCTTTTATATTC-3′. Then the PCR products were cloned into pET19b vector (Novagen, Madison, WI, USA). For recombinant protein expression, the cloned DNA was transformed into *E. coli* strain BL21-CodonPlus (DE3) RIL (Agilent Technologies, Santa Clara, Ca, USA). Expression of r0190 was induced in Luria-Bertani broth in the presence of 0.4 mM isopropyl-β-D-thiogalactopyranoside (IPTG) (Amresco, Solon, OH, USA) for 12 h at 28 °C. The r0190 protein was purified by Fast Protein Liquid Chromatography (FPLC) using Ni-NTA affinity columns (Qiagen, Hilden, Germany). For production of immune serum against r0190, BALB/c mice were immunized 3 times every 2 weeks with 20 μg of purified r0190 protein. Two weeks after last immunization, blood samples from immunized mice were collected and used for the experiments including Western blot and immunoelectron microscopy.

### Cell culture and stimulation

Bone marrow cells were isolated from TLR2-WT and TLR2-KO female mice as described previously [36]. For differentiation of BMDMs, bone marrow cells were cultured in DMEM with 50 μM β-mercaptoethanol in the presence of 30% L929 conditioned media for 6 to 8 days at 37 °C in a humidified incubator with 5% CO_2_. For ELISA experiments, BMDMs were seeded in 24-well plates at 1 × 10^6^ cells/mL and stimulated with r0190 (0.1, 0.3, or 1 μg/ml), P3C (0.1 or 0.5 μg/ml), or CpG (0.1 μM) for 24 h. HEK293-TLR2 cells were incubated in DMEM in the presence of blasticidin (10 μg/ml). The cells were preincubated with isotype control IgG1, anti-TLR1 (5 μg/ml), or anti-TLR6 for 1 h and then stimulated with r0190 (1 μg/ml), P3C (0.1 μg/ml), or MALP2 (0.01 μg/ml) for an additional 24 h.

### ELISA and Western blot analysis

Mouse TNF-α, IL-6, MCP-1, MIP-1α, and human IL-8 concentrations were estimated by ELISA kits according to the manufacturer’s protocols. Thirty micrograms of *B. anthracis* protein fractions, including spore lysates, vegetative cell proteins, and secreted proteins, were purified as described previously [37, 38], separated by SDS-PAGE and then probed with normal mouse serum or r0190 immune serum. For Western blot analysis, BMDMs were treated with 1 μg/ml of r0190 for the indicated time periods. Then the cells were lysed and their lysates were subjected to Western blot analysis as described previously [36].

To determine specific IgG antibody levels against PA or GBAA0190, ELISAs were performed as previously described with some modifications [39]. In brief, 96 well ELISA plates (Nunc, Roskilde, Denmark) were coated with PA (100 ng/well) or r0190 (100 ng/well) overnight at 4 °C and then incubated with sera diluted from 1:20 to 14,580 for 1 h at 37 °C. Horseradish peroxidase-conjugated goat anti-guinea pig IgG antibody (Sigma-Aldrich) and 3,3`,5,5`-tetramethylbenzidine substrate (Biolegend) were for detection. The optical density (OD) of each well was measured at 450 nm and ELISA titers were calculated using a cutoff absorbance value of average background OD + 3 SD (standard deviation). The data were analyzed using GraphPad Prism 6.

### Immunofluorescence and Immunoelectron microscopy

BMDMs (1 × 10^5^/ml) were plated on a chamber slide (Nunc, Roskilde. Denmark) and stimulated with 1 μg/ml r0190 for 1 h. Immunofluorescence microscopy was conducted to examine NF-κB activation by r0190 as described previously [22]. *B. anthracis* H9401 spores were fixed with fixation solution (Biolegend, San Diego, CA, USA) for 24 h at 4 °C and washed with phosphate-buffered saline (PBS). Then, they were resuspended in periodate-paraformaldehyde-lysine-sucrose (periodate 0.2%, lysine 1.4%, sucrose 15%) solution. Spore solutions were loaded on 200 mesh nickel grids and dried by air for 1 h. Then, grids were washed with PBS and 3% bovine serum albumin (w/v) in PBS was treated on the grids for 20 min. Mouse r0190 serum was diluted in PBS (1:3000) and loaded on the grids for 1 h. Subsequently, the grids were washed with PBS and incubated with anti-mouse IgG antibodies conjugated to 10 nm gold particle (Abcam, Eugene, OR, USA) at a dilution of 1:50 for 1 h. After PBS washing and air drying, photographs were taken on a ZEISS LIBRA 120 transmission electron microscope at an accelerating voltage of 120 kV and at magnifications of 31,500-fold. Normal mouse serum was tested as a control.

### Antigen immunization and *B. anthracis* spore challenge

All the GPs were divided into 4 groups which were composed of 6 animals in each group. Group 1 was immunized with rPA (5 μg) and group 2 received r0190 (5 μg). Group 3 was vaccinated with both rPA (5 μg) and r0190 (5 μg). Each vaccine was immunized by mixing with alum (25 μg/ml), while group 4 received alum alone. GPs were immunized with freshly prepared antigen mixtures by intramuscular route (i.m.) on day 0 and given a boost on day 28 as described previously [40]. To assess the protective activity of each antigen, immunized GPs were challenged with 15 or 30 LD_50_ of *B*. *anthracis* H9401 spores on day 56 and monitored for 15 days.

### Statistical analysis

Differences in survival between groups of GPs were determined using the log-rank test with GraphPad Prism 6.0 software (GraphPad Software, Inc., San Diego, CA, USA). For other measures, the mean values ± SD were determined for each treatment group in the individual experiment. Treatment groups were compared with the appropriate control, and statistical significance was calculated with the two-tailed Student’s *t*-test or multiple-comparison test after one-way ANOVA. Differences were considered significant when the *P* value was < 0.05.

## Supplementary Information

**Additional file 1.**

## Data Availability

The datasets used and/or analyzed during the current study are available from the corresponding author on reasonable request.

## Abbreviations

r0190: Recombinant GBAA0190
APC: Antigen presenting cells
PA: Protective antigen
EF: Edema factor
LF: Lethal factor
LT: Lethal toxin
ET: Edema toxin
TNF-α: Tumor necrosis factor-α
IL: Interleukin
MCP-1: Monocyte chemoattractant protein-1
MIP-1α: Macrophage inflammatory protein-1α
cAMP: Cyclic adenosine monophosphate
MAP: Mitogen-activated protein
NF-κB: Nuclear factor-κB
Lgt: Prolipoprotein diacylglyceryl transferase
SDS-PAGE: Sodium dodecyl sulfate-polyacrylamide gel electrophoresis
MALP2: Macrophage-activating lipopeptide2
AVA: Anthrax vaccine adsorbed
AVP: Anthrax vaccine precipitated
TLR: Toll-like receptor
GP: Guinea pig
PA-FIS: PA plus formaldehyde-inactivated spores
P3C: PAM3CSK4
CpG: CpG2395
ELISA: Enzyme-linked immunosorbent assay
WT: Wild-type
KO: Knockout
IPTG: Isopropyl-β-D-thiogalactopyranoside
FPLC: Fast Protein Liquid Chromatography
BMDMs: Bone marrow-derived macrophages
SL: Spore lysates
VCP: Vegetative cell proteins
SP: Secreted proteins
CBB: Coomassie brilliant blue
IM: Intramuscular
Alum: Aluminum hydroxide
OD: Optical density
SD: Standard deviation
