# Supplementary material for: A putative exosporium lipoprotein GBAA0190 of Bacillus anthracis as a potential anthrax vaccine candidate
Source: BMC Immunol. 2021 Mar 21;22:20. doi: 10.1186/s12865-021-00414-y (PMC7981958; doi:10.1186/s12865-021-00414-y)
Supplement: Supplementary file 1 — Additional file 1. [file 12865_2021_414_MOESM1_ESM.pptx]

## Slide 1
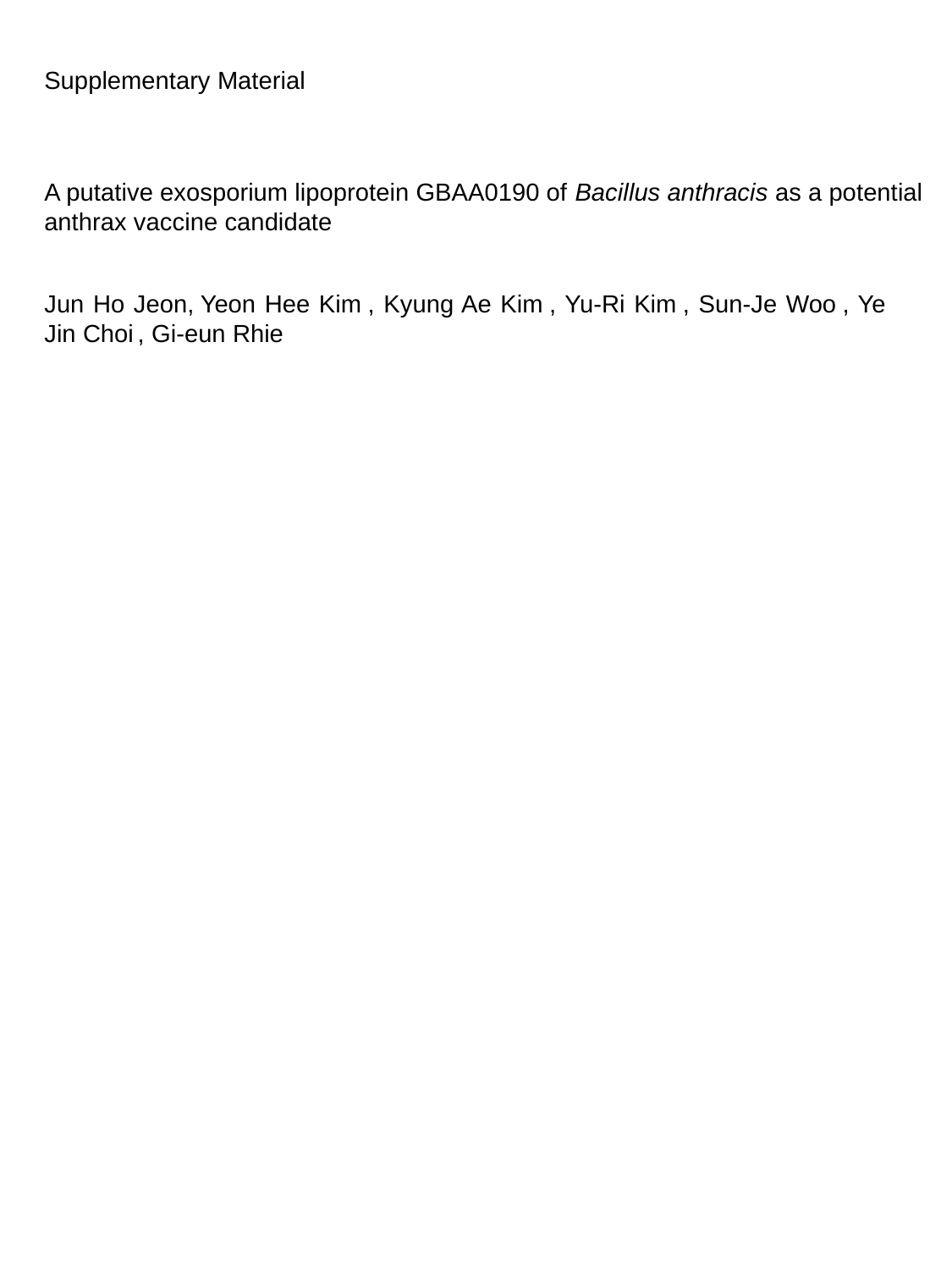

Supplementary Material
A putative exosporium lipoprotein GBAA0190 of Bacillus anthracis as a potential anthrax vaccine candidate
Jun Ho Jeon, Yeon Hee Kim , Kyung Ae Kim , Yu-Ri Kim , Sun-Je Woo , Ye Jin Choi , Gi-eun Rhie

## Slide 2
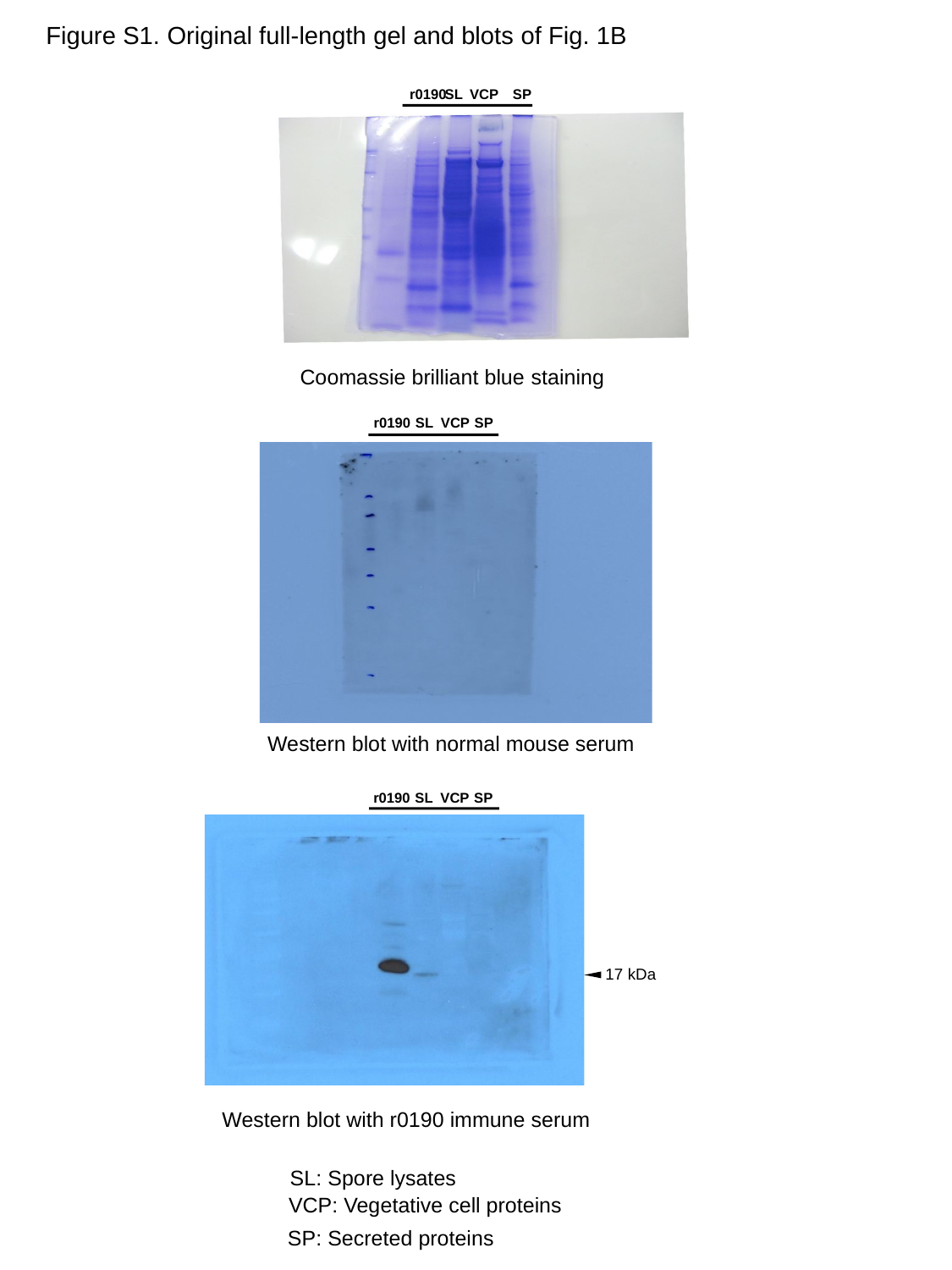

Figure S1. Original full-length gel and blots of Fig. 1B
r0190
SL
 VCP
 SP
Coomassie brilliant blue staining
r0190
SL
 VCP
 SP
Western blot with normal mouse serum
r0190
SL
 VCP
 SP
17 kDa
Western blot with r0190 immune serum
 SL: Spore lysates
VCP: Vegetative cell proteins
SP: Secreted proteins

## Slide 3
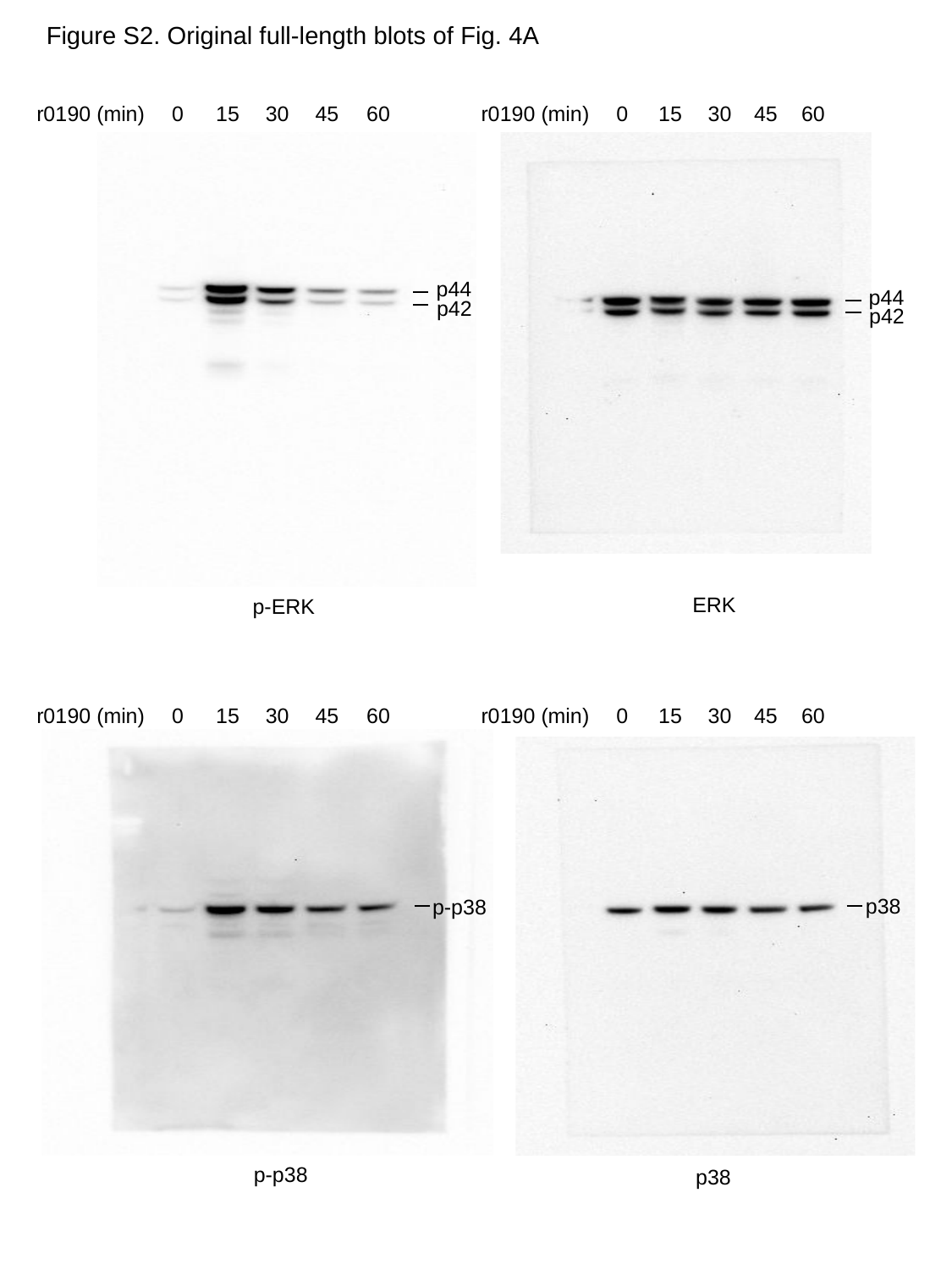

Figure S2. Original full-length blots of Fig. 4A
r0190 (min)
0
15
30
45
60
r0190 (min)
0
15
30
45
60
p44
p44
p42
p42
ERK
p-ERK
r0190 (min)
0
15
30
45
60
r0190 (min)
0
15
30
45
60
p38
 p-p38
p-p38
p38

## Slide 4
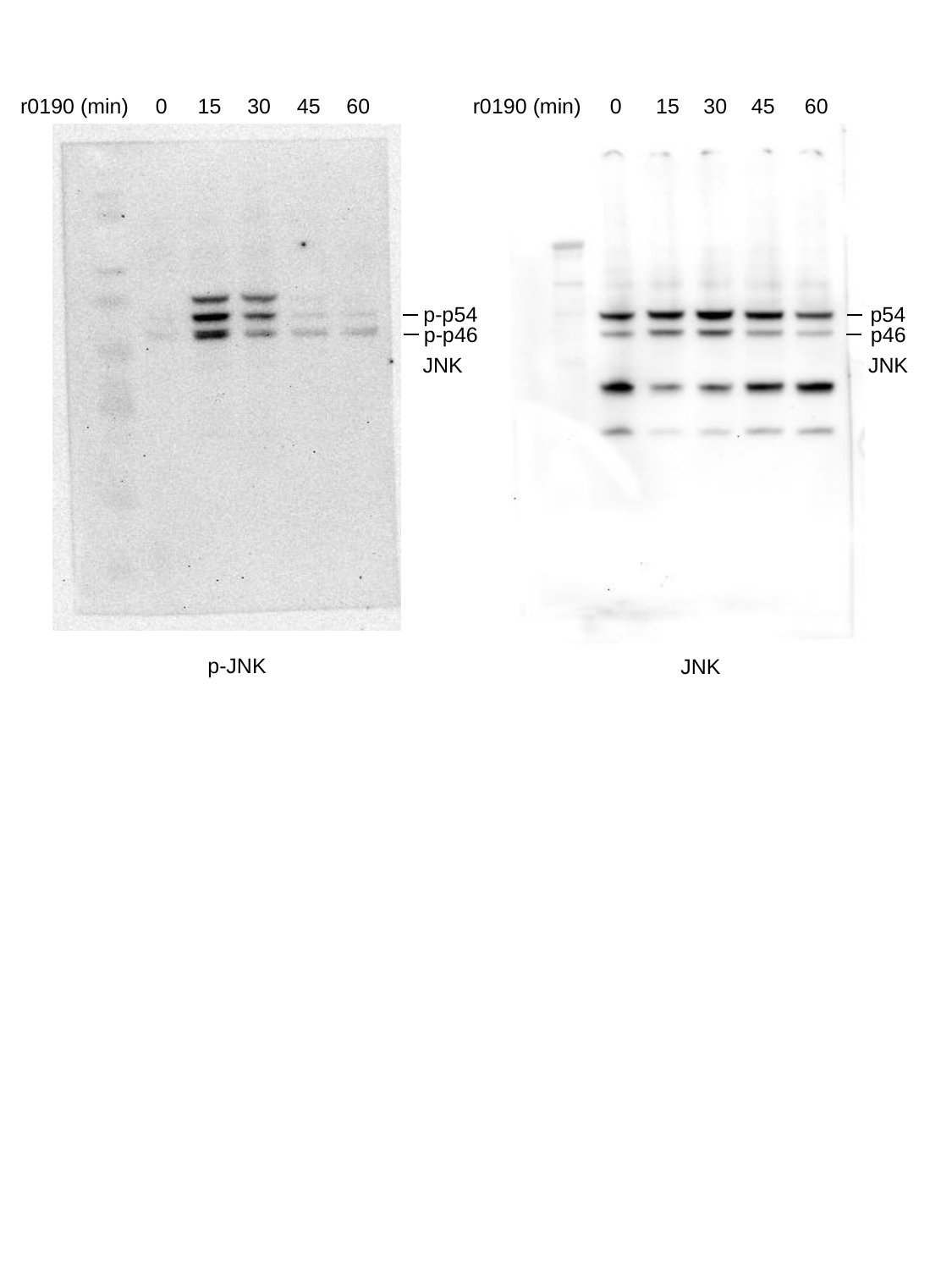

r0190 (min)
0
15
30
45
60
r0190 (min)
0
15
30
45
60
p-p54
p54
p-p46
p46
JNK
JNK
p-JNK
JNK

## Slide 5
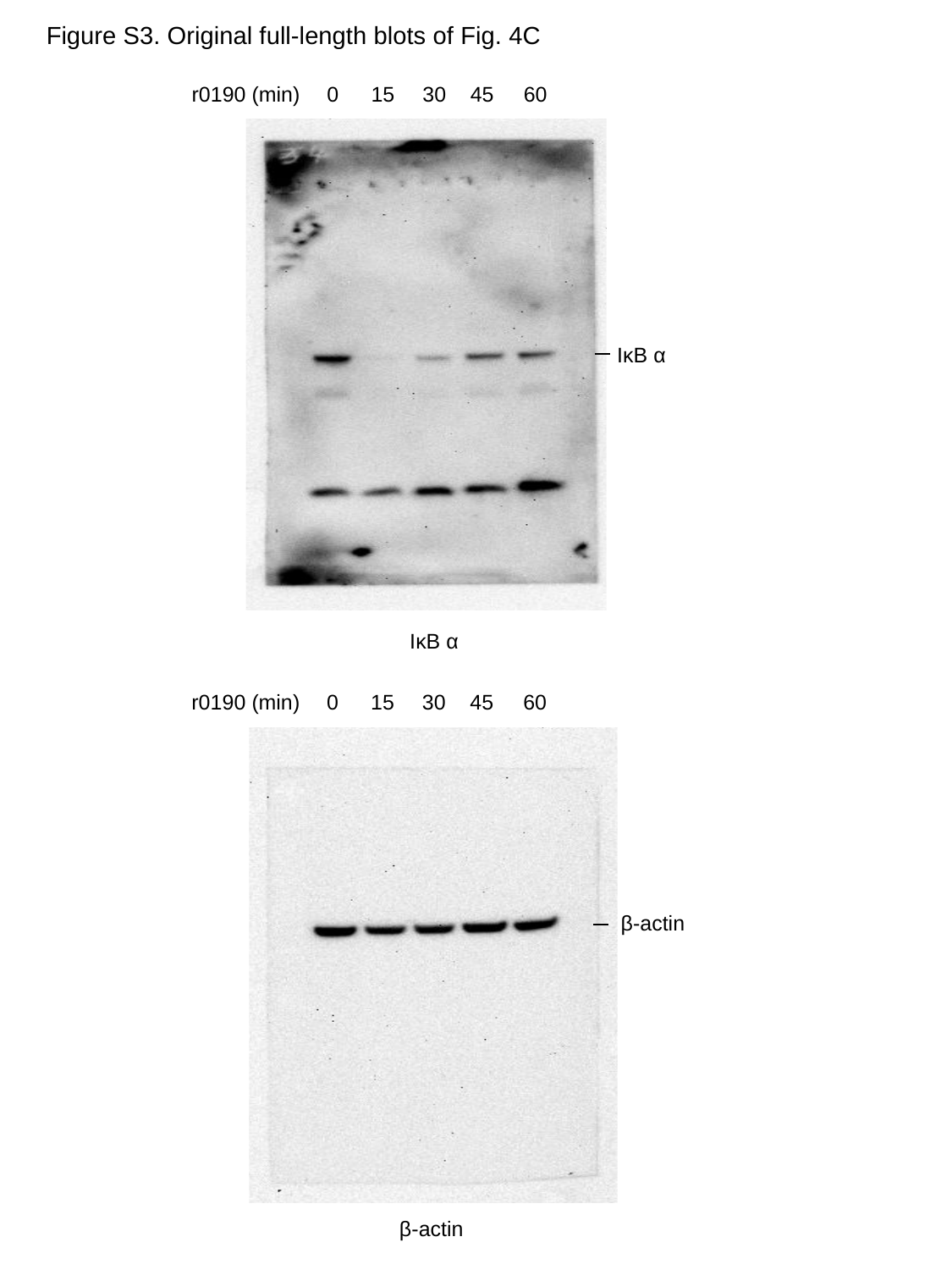

Figure S3. Original full-length blots of Fig. 4C
r0190 (min)
0
15
30
45
60
IκB α
IκB α
r0190 (min)
0
15
30
45
60
β-actin
β-actin
